# Predictive factors for HIV infection among men who have sex with men and who are seeking PrEP: a secondary analysis of the PROUD trial

**DOI:** 10.1136/sextrans-2018-053808

**Published:** 2019-03-27

**Authors:** Ellen White, David T Dunn, Monica Desai, Mitzy Gafos, Peter Kirwan, Ann K Sullivan, Amanda Clarke, Sheena McCormack, PROUD study group, David I Dolling

**Affiliations:** 1 Medical Research Council Clinical Trials Unit at University College London, London, UK; 2 National Institute for Health and Care Excellence, Manchester, UK; 3 Department of Global Health and Development, London School of Hygiene and Tropical Medicine, London, UK; 4 HIV and STI Department, National Infection Service, Public Health England, London, UK; 5 St Stephen's Centre, Chelsea and Westminster Hospital NHS Foundation Trust, London, UK; 6 Department of HIV, Sexual Health and Contraception, Royal Sussex County Hospital, Brighton and Sussex University Hospitals NHS Trust, Brighton, UK; 7 Brighton and Sussex Medical School, University of Sussex, Brighton, UK; 8 56 Dean Street, Chelsea and Westminster Hospital NHS Foundation Trust, London, UK

**Keywords:** HIV, MSM, PrEP, eligibility criteria

## Abstract

**Objectives:**

Pre-exposure prophylaxis (PrEP) is a highly effective method of HIV prevention for men who have sex with men (MSM). However, uncertainty remains around the optimal eligibility criteria for PrEP, specifically whether there are subgroups at low risk of HIV for whom PrEP might not be warranted.

**Methods:**

PROUD was an open-label waitlist trial design that randomised MSM attending participating sexual health centres in England to receive PrEP immediately (IMM) or after a deferral period of 1 year (DEF). This analysis is based on participants who were randomised to the deferred arm, when they did not have access to PrEP. HIV incidence was compared between subgroups defined by baseline characteristics.

**Results:**

Overall, 21 participants acquired HIV infection over 239.3 person-years (PY) follow-up, yielding an incidence rate of 8.8/100 PY (95% CI 5.4 to 13.4). Two highly significant predictors for HIV acquisition were identified. Men with a self-reported diagnosis of syphilis, rectal chlamydia (CT) or rectal gonorrhoea (GC) in the previous 12 months had an incidence of 17.2/100 PY (95% CI 9.7 to 28.5); those reporting receptive anal intercourse without a condom (ncRAI) with two or more partners in the previous 3 months had an incidence of 13.6/100 PY (95% CI 7.9 to 21.7). The incidence rate among participants lacking both of these risk factors was 1.1/100 PY (1/87.6, 95% CI 0.03 to 6.4).

**Conclusions:**

The high HIV incidence in PROUD suggests that most participants appropriately judged their need for PrEP. Eligibility criteria for a PrEP programme can therefore be broad, as in the current guidelines. However, a recent history of syphilis or rectal CT/GC, or multiple ncRAI partners indicates a high imminent risk of HIV infection. MSM with any of these characteristics should be offered PrEP as a matter of urgency.

## Introduction

Oral pre-exposure prophylaxis (PrEP) has been shown to be a highly effective method of HIV prevention for men who have sex with men (MSM).^1–3^ At a population level, this effect is mediated both through the direct avoidance of infections among individuals who take PrEP and the indirect prevention of infections that would have occurred subsequently in the transmission chain.^4^ In the UK, new HIV diagnoses among MSM remained stable for over a decade but have declined in the last 2 years, likely mainly as a result of the combination of PrEP, an increase in HIV testing and routine rapid treatment after diagnosis.^5 6^

An important issue in PrEP roll-out programmes is participant eligibility since cost-effectiveness is critically dependent on the HIV incidence in the target population. From a clinical perspective the risk:benefit ratio of PrEP may be disadvantageous in individuals at negligible risk of HIV infection, for example, those in a monogamous serodifferent relationship whose partner is virologically suppressed on treatment. WHO guidelines recommend PrEP in populations with an annual incidence greater than 3%.^7^ However, individual risk of acquiring HIV infection is highly heterogeneous and implementation of this recommendation is not straightforward. Several organisations have issued PrEP guidelines, which include eligibility criteria for MSM.^8–10^ The central criterion in all guidelines is reported anal intercourse without a condom, although with no explicit reference to the number of partners. Other criteria include the use of postexposure prophylaxis (PEP), a recent diagnosis with a bacterial STI and a history of sexualised drug use.

PROUD was an open-label trial that randomised MSM attending participating sexual health centres in the England to receive PrEP immediately or after a deferral period of 1 year (waitlist design).^2 11^ A key finding from the trial was the unexpectedly high HIV incidence during the deferral period (9 per 100 person-years [PY]). Here we present an analysis of baseline risk factors for the acquisition of HIV among men randomised to the deferred group before they had access to PrEP. The objective of this analysis was to examine the relative importance of the recommended eligibility criteria for a future PrEP programme, identify any other risk factors and explore whether there are subgroups at low risk of HIV for whom PrEP might not be warranted. The results should inform eligibility criteria for MSM in the UK and similar populations elsewhere in Europe.

## Methods

Participants were enrolled into the PROUD trial between November 2012 and April 2014, and were randomly assigned to start PrEP immediately at enrolment (IMM) or after a 1-year deferral period (DEF). Eligible participants were HIV negative; tested by a routinely used assay in the previous 4 weeks or on the day of enrolment, male at birth, aged 18 years or older, had attended the enrolling clinic previously, and reported anal sex without a condom in the previous 90 days and stated it likely that this behaviour would recur again in the next 90 days. At enrolment, participants’ self-completed a questionnaire on: demographic characteristics, including age, education, country of birth, whether they had been circumcised and relationship status; self-reported history of number of HIV/STI screens, STIs and the use of post-exposure prophylaxis (PEP) in the previous 12 months; and sexual behaviour in the previous 90 days. Sexual behaviour included the use of poppers and drugs most commonly associated with chemsex (defined in the footnote to table 1), and the number of different anal sex partners (total, receptive sex, insertive sex, receptive/insertive sex without a condom, receptive/insertive sex without a condom and partner known to be HIV positive). Follow-up visits, including a full STI screen, were scheduled every 3 months.

**Table 1:** HIV incidence by baseline characteristics

Characteristic		Participants, n (%)	Total PY	HIV infections	Incidence rate (per 100 PY)	95% CI	Rate ratio	95% CI	P value*
Total		268 (100.0)	239.3	21	8.8	5.4 to 13.4	–	–	–
Age (years)	18–24	27 (10.1)	24.6	3	12.2	2.5 to 35.7	1.4	0.3 to 4.8	0.26
	25–34	104 (38.8)	94.5	9	9.5	4.4 to 18.1	1.1	0.4 to 2.8	
	35–49	116 (43.3)	101.0	9	8.9	4.1 to 16.9	1.0	–	
	50+	21 (7.8)	19.1	0	0	0 to 19.3†	0.4	0 to 2.1†	
University degree	No	101 (38.0)	90.6	6	6.6	2.4 to 14.4	1.0	–	0.38
	Yes	165 (62.0)	146.7	15	10.2	5.7 to 16.9	1.5	0.6 to 4.3	
Full-time employment	No	68 (25.8)	63.1	3	4.8	1.0 to 13.9	1.0	–	0.20
	Yes	196 (74.2)	173.0	18	10.4	6.2 to 16.4	2.2	0.7 to 9.3	
Born in UK	No	107 (40.2)	96.6	9	9.3	4.3 to 17.7	1.0	–	0.84
	Yes	159 (59.8)	140.7	12	8.5	4.4 to 14.9	0.9	0.4 to 2.3	
Ethnicity	White	218 (82.6)	192.3	19	9.9	5.9 to 15.4	1.0	–	0.32
	Black, Asian and minority	46 (17.4)	42.8	2	4.7	0.6 to 16.9	0.5	0.07 to 1.8	
London site	No	81 (30.2)	71.8	6	8.4	3.1 to 18.2	1.0	–	0.91
	Yes	187 (69.8)	167.4	15	9.0	5.0 to 14.8	1.1	0.4 to 3.0	
Circumcised	No	185 (70.1)	166.2	17	10.2	6.0 to 16.4	1.0	–	0.30
	Yes	79 (29.9)	69.8	4	5.7	1.6 to 14.7	0.6	0.2 to 1.6	
Relationship status	Living with partner	73 (27.5)	66.9	6	9.0	3.3 to 19.5	0.9	0.3 to 2.3	0.32
	Not living with partner	46 (17.4)	42.1	2	4.7	0.6 to 17.2	0.5	0.07 to 1.8	
	Single	146 (55.1)	127.3	13	10.2	5.4 to 17.5	1.0	–	
High depression score‡	No	233 (92.1)	205.1	19	9.3	5.6 to 14.5	1.0	–	0.68
	Yes	20 (7.9)	18.5	1	5.4	0.1 to 30.2	0.6	0.03 to 3.2	
Number of HIV tests§	0–2	91 (35.5)	82.4	5	6.1	2.0 to 14.2	0.7	0.2 to 2.1	0.13
	3–4	122 (47.7)	106.1	9	8.5	3.9 to 16.1	1.0	–	
	5+	43 (16.8)	38.1	6	15.7	5.8 to 34.2	1.9	0.6 to 5.3	
Key STI§	No	155 (60.5)	140.4	5	3.6	1.2 to 8.3	1.0	–	0.001
	Rectal CT/GC or syphilis	101 (39.5)	87.0	15	17.2	9.7 to 28.5	4.8	1.8 to 14.9	
PEP use§	No	159 (63.3)	142.3	11	7.7	3.9 to 13.8	1.0	–	0.41
	Yes	92 (36.7)	80.3	9	11.2	5.1 to 21.3	1.4	0.6 to 3.6	
Number of ncRAI partners¶	0	32 (12.5)	29.8	1	3.4	0.08 to 18.7	1.2	0.04 to 16.0	0.01
	1	78 (30.5)	72.5	2	2.8	0.3 to 10.0	1.0	–	
	2–4	81 (31.6)	68.7	8	11.6	5.0 to 22.9	4.2	1.0 to 29.1	
	5–9	35 (13.7)	30.9	5	16.2	5.3 to 37.8	5.9	1.2 to 43.7	
	10+	30 (11.7)	25.7	4	15.6	4.2 to 39.9	5.7	1.0 to 44.1	
Drug use associated with chemsex¶**	No	135 (52.3)	121.9	7	5.7	2.3 to 11.8	1.0	–	0.17
	Yes	123 (47.7)	108.1	12	11.1	5.7 to 19.4	1.9	0.8 to 5.2	
Poppers¶	No	129 (50.0)	117.9	10	8.5	4.1 to 15.6	1.0	–	0.91
	Yes	129 (50.0)	112.1	9	8.0	3.7 to 15.2	0.9	0.4 to 2.4	

Missing data (total, events lost due to missing exposure data) for education (2, 0); employment status (4, 0); born in UK (2, 0); ethnicity (4, 0); circumcised (4, 0); relationship (3, 0); depression (15, 1); number of HIV tests (12, 1); key STI (12, 1); PEP (17, 1); ncRAI (12, 1); chemsex (10, 2); poppers (10, 2).

Quantitative variables were grouped according to clinical considerations.

Key STIs (rectal chlamydia [CT], rectal gonorrhoea [GC] or syphilis).

*P value for trend calculated for ordered categorical variables: ncRAI partners, anal intercourse (AI) partners, age and HIV tests. P value for relationship status compares single versus not living with partner. P value for ethnicity compares white against all other categories combined.

†One sided, 97.5% CI.

‡Defined by the Patient Health Questionnaire-9 (PHQ-9) score, high score ≥10.

§Occurred in 12 months prior to baseline visit.

¶Occurred in the 90 days prior to baseline visit.

**Chemsex-associated drugs defined as the use of methamphetamine, gamma hydroxybutyrate (GHB), mephedrone or ketamine.

PEP, postexposure prophylaxis; PY, person-years; ncRAI, receptive anal intercourse without a condom.

This analysis is based on participants who were randomised to the DEF arm during the period when they did not have access to PrEP. An incident HIV infection was defined as a reactive HIV antigen-antibody test result, confirmed by the detection of HIV RNA, in participants who were HIV negative at enrolment. Originally, PrEP was planned to be offered at the 12-month scheduled visit. However, in October 2014, based on an interim analysis, the Independent Data Monitoring Committee recommended that all participants should be offered PrEP. Loss to follow-up in the trial was low, with HIV status known in 89% of participants at the end of the deferred phase.^2^ We additionally sought to identify participants who may have been diagnosed with HIV in a non-study clinic by linking (based on pseudoanonymised identifiers) to the Public Health England (PHE) national database of new HIV diagnoses.^12^

HIV incidence was compared between different subgroups according to baseline data. For participants who acquired HIV, date of infection was taken to be the date of the first reactive test. Follow-up was censored at the first visit either when a participant was actually offered PrEP (if they remained in follow-up) or when a participant would have been offered PrEP within the trial (if they experienced early loss to follow-up). This differs from the censoring rules in the main analysis, which were based on the timing of HIV tests in study clinics and did not consider PHE linkage data. In addition, two participants enrolled twice in the trial (first to DEF and then to IMM). In the main analysis, their entire follow-up was assigned according to their original randomisation allocation (intention to treat); in the present analysis, their follow-up is censored at the date of their second randomisation (when they were offered PrEP).^2^ Online supplementary appendix table 1 summarises the censoring rules described above.

10.1136/sextrans-2018-053808.supp1 Supplementary data

Due to the small number of events, we used exact Poisson methods (*ci* command for incidence rates [IR] and *expoisson* for rate ratios [RR] with the *midp* option). For categorical variables, the most frequent category was used as the reference group. To examine whether incidence changed over the period of follow-up, a Weibull model was compared against an exponential model. Statistical analyses were conducted in Stata V.14.0.

## Results

A total of 268 participants from the DEF arm were included in the analysis after excluding one individual due to a reactive HIV antigen-antibody test at enrolment, despite a non-reactive point-of-care test. Baseline questionnaires were not completed by two individuals and responses were occasionally missing for some questions for others (maximum 6.3% missing, table 1, footnote). At enrolment, median age was 35 years (IQR: 28–41), 40.2% were born outside the UK, 69.8% were recruited through a London clinic and 55.1% were single (table 1). In the previous 12 months, 39.5% reported they had been diagnosed with rectal chlamydia (CT), rectal gonorrhoea (GC) or syphilis (referred to subsequently as a key STI), and 36.7% had received at least one PEP prescription. There was wide variability in the reported number of anal sex partners in the 90 days prior to enrolment. 9.2% reported a single partner while 27.5% reported 20 or more with a median of 10 (IQR: 4–20). A high proportion reported receptive anal intercourse without a condom (ncRAI); 87.5% at least once and 11.7% reported 10 or more such partners.

Overall, 21 participants acquired HIV infection over 239.3 PY follow-up, yielding an IR of 8.8 per 100 PY (95% CI 5.4 to 13.4 per 100 PY). Matching with the national HIV new diagnoses database yielded an additional 19.4 years of follow-up (from 31 participants), and one further HIV infection in addition to the 20 infections originally reported.^2^ During the relatively short period of follow-up, there was a non-significant (p=0.13) increase in HIV incidence (Weibull shape parameter=1.4).

Reporting a diagnosis of a key STI in the previous 12 months was a highly significant predictor for HIV acquisition. The IR in this subgroup was 17.2 per 100 PY (15/87.0, 95% CI 9.7 to 28.5), 4.8-fold higher (95% CI 1.8 to 14.9) than the rate for men who did not report such a diagnosis. Incidence did not vary substantially according to the specific key STI that was reported (table 2). Table 2 also shows that HIV incidence was relatively low among participants reporting a pharyngeal or urethral STI (without a rectal STI).

**Table 2:** Associations between STIs and HIV incidence rate

	Participants, n (%)	Total PY	HIV infections (n)	Incidence rate (per 100 PY)	95% CI
Rectal CT/GC or syphilis (key STI)	101 (39.4)	87.0	15	17.2	9.7 to 28.5
Syphilis	30 (11.7)	24.0	5	20.8	6.8 to 48.6
Rectal CT/GC	83 (32.4)	72.9	12	16.5	8.5 to 28.8
Rectal CT	56 (21.9)	49.8	8	16.1	6.9 to 31.6
Rectal GC	62 (24.2)	54.0	8	14.8	6.4 to 29.2
Excluding participants reporting rectal infection or syphilis					
Pharyngeal infection	25 (16.1)	23.5	0	0	0 to 15.7*
Urethral infection	33 (21.3)	30.6	1	3.3	0.08 to 18.2

*One sided, 97.5% CI.

GC, gonorrhoea CT, chlamydia; PY, person-years.

The other strong predictive factor was the number of self-reported anal sex partners in the previous 90 days, whether this was expressed overall, just for receptive sex or further limited to condomless sex (online supplementary appendix table 2). These variables are highly correlated and therefore difficult to distinguish, although the clearest gradient in risk was seen for the number of ncRAI partners. A threshold effect was evident, with HIV risk sharply elevated for men reporting two or more partners; HIV incidence in this subgroup was 13.6 per 100 PY (17/125.3, 95% CI 7.9 to 21.7), with an RR of 4.6 (95% CI 1.5 to 19.8). Thirty-one participants (12.2%) reported that the only condomless sex they had was insertive, none of whom acquired HIV during follow-up (95% CI 0 to 12.3 per 100 PY); 16 (51.6%) of these participants were circumcised.

The diagnosis of a key STI in the year prior to enrolment was closely related to the number of ncRAI partners in the previous 3 months. For those with 0, 1, 2–4, 5–9 and 10+partners, 22.6%, 30.7%, 40.0%, 54.5% and 66.7% reported an STI in the previous year, respectively (p value for trend <0.001). In a bivariate model that included both ncRAI and key STI, the relative risk estimates for two or more ncRAI partners were attenuated (RR 4.6 to 2.9) but the estimate for the diagnosis of a key STI was largely unchanged (RR 4.8 to 4.7). All but one (20/21, 95.2%) of the HIV infections occurred among the 177 participants who reported either a key STI or ncRAI with two or more partners; the PY observed in the group reporting either of these characteristics comprised 63.4% (151.6/239.3) of the overall follow-up. The IR of the participants lacking both of these risk factors was 1.1 per 100 PY (1/87.6, 95% CI 0.03 to 6.4 per 100 PY).

Non-significant trends in the expected direction were seen for other variables, including PEP use, use of chemsex-associated drugs, the number of HIV tests age (younger men at higher risk) (table 1). Participants in full-time employment were at higher risk of HIV compared to those not in full-time employment. There was no effect of location of clinic (London vs non-London) or whether the participant was born in the UK. Participants in a relationship but who were not cohabiting were at a lower risk of HIV infection than either single men or men in a cohabiting relationship.

## Discussion

The most powerful individual predictor of HIV infection in PROUD was the diagnosis of syphilis or a bacterial rectal infection (CT/GC) in the previous year; HIV incidence was 17.2 per 100 PY in this subpopulation. A recent analysis of MSM who were repeat attenders at genitourinary medicine (GUM) clinics in England identified similar factors but the strength of the association was weaker and the proportion of infections associated with this risk factor was much smaller.^13^ This is likely explained by the lower HIV incidence and smaller proportion of attenders with a history of an STI in the national study. A secondary analysis of the iPrEx trial examined predictors of HIV infection in the placebo arm.^14^ The only STI variable reported in this trial was whether an infection had been reported in the previous 6 months, irrespective of the type or site of infection. This lack of specificity may explain the small difference in HIV incidence between participants with (4.9 per 100 PY) and without (3.6 per 100 PY) an STI report. The strong effect in PROUD may be partly due to the regularity of STI screening in sexual health clinics in the UK, with participants receiving a median of three screens in the year prior to enrolment.^11^ Pharyngeal and urethral STIs were not associated with an increased HIV risk, suggesting that the selection for variables to include in risk algorithms should focus on rectal infections and early syphilis.

The other important predictive variable was the number of sexual partners in the previous 3 months, particularly the number of ncRAI partners. Participants with fewer than two ncRAI partners were at a comparatively lower risk of HIV infection (2.9 per 100 PY), falling just below the WHO threshold of ‘substantial’ risk. The risk fell further below this threshold when combined with the absence of a key STI diagnosis, suggesting that this subgroup should be a lower priority to receive PrEP (ie, one or fewer ncRAI partners in previous 3 months *and* no key STI diagnosed in previous 12 months). In the extreme case where PrEP confers 100% protection, 95% of incident HIV infections would have been prevented by providing PrEP only to individuals reporting *either* a key STI *or* ncRAI with two or more partners (63% of the PY of follow-up). While the very high HIV incidence in PROUD raises the question of generalisability to other lower risk settings, we believe this concern applies more to our quantitative estimates than to the findings in general, which should be broadly applicable. Another clinically relevant finding was the low risk for men who engage exclusively in condomless insertive anal intercourse, mirroring findings in the iPrEx study.^14^ However, individual clinical decisions clearly should take into account current risk behaviours as well as historical and anticipated ones. The definition of ‘historical’ is also not standardised across guidelines, some referring to the previous 6 months and some not specifying a time frame at all.^8 9^

HIV incidence among PROUD participants without access to PrEP was fourfold higher than that of the MSM population attending GUM clinics in the UK.^2 13^ This is likely to represent an enrichment phenomenon where self-awareness of a high risk of acquiring HIV infection motivates individuals to seek PrEP.^15^ If genuine, this phenomenon is favourable for the cost-effectiveness of PrEP, which is highly dependent on HIV incidence in the group to whom it is offered.^16 17^ Analyses of risk factors for the acquisition of HIV infection have been conducted in cohort studies of MSM in several countries,^13 18 19^ often with a motivation to inform eligibility criteria for PrEP or to identify individuals at particularly high risk who could potentially be targeted.^14 18^ A limitation of these studies was an inability to identify those participants who would have been interested in taking PrEP had it been available. The key strength of this secondary analysis of PROUD is the restriction to MSM who were either actively seeking PrEP or had accepted a clinical recommendation from their clinician.

The main limitation of our analysis is the low number of incident HIV infections, but this was unavoidable due to the size of the trial. The consequences are twofold: imprecise estimates of HIV incidence and an inability to undertake multivariate analyses to develop a risk algorithm. Although PrEP was not available through the National Health Service during the study period, some participants in the deferred arm may have accessed it through other means. PEP use was also common with 174 courses prescribed to 85 participants during the study period,^2^ which could have further reduced the estimated incidence. Finally, our results are sensitive to the specific recall periods that were used in the questionnaires.

In conclusion, the high HIV incidence in PROUD suggests that participants appropriately judged their risk of acquiring HIV and the benefits of PrEP. Eligibility criteria for a PrEP programme following PROUD can therefore be broad for MSM, as in the current guidelines. However, the risk of acquiring HIV is declining in some settings due to increased PrEP coverage, an increase in HIV testing and rapid treatment after an HIV diagnosis.^6 20^ This complicates this judgement and may change the predictive value of individual risk factors. In particular, we note a possible further reduction in the already low risk for those reporting only one partner with whom they had not used a condom for receptive anal sex and it will be important to continue to review the need for PrEP in this group. However, a recent history of syphilis, rectal CT/GC or two or more ncRAI partners indicates a high imminent risk of HIV infection, and HIV-negative MSM with any of these characteristics should be offered PrEP as a matter of urgency.

**Key messages**

- An important unresolved question in pre-exposure prophylaxis (PrEP) programmes is patient eligibility.
- As the amount of PrEP is generally limited, it is important that it is offered to those at the highest risk of acquiring HIV.
- Highest HIV risk was among men who have sex with men (MSM) reporting a recent rectal STI or syphilis diagnosis, or reporting condomless receptive anal intercourse with two or more partners.
- HIV-negative MSM with either of these characteristics should be offered PrEP as a matter of urgency.

## References

[1] Grant RM, Lama JR, Anderson PL, et al Preexposure chemoprophylaxis for HIV prevention in men who have sex with men. N Engl J Med Overseas Ed 2010;363:2587–99. 10.1056/NEJMoa1011205 PMC3079639 21091279

[2] McCormack S, Dunn DT, Desai M, et al Pre-exposure prophylaxis to prevent the acquisition of HIV-1 infection (PROUD): effectiveness results from the pilot phase of a pragmatic open-label randomised trial. The Lancet 2016;387:53–60. 10.1016/S0140-6736(15)00056-2 PMC4700047 26364263

[3] Molina J-M, Capitant C, Spire B, et al On-demand preexposure prophylaxis in men at high risk for HIV-1 infection. N Engl J Med 2015;373:2237–46. 10.1056/NEJMoa1506273 26624850

[4] Jenness SM, Goodreau SM, Rosenberg E, et al Impact of the Centers for disease control's HIV preexposure prophylaxis guidelines for men who have sex with men in the United States. J Infect Dis 2016;214:1800–7. 10.1093/infdis/jiw223 27418048 PMC5142082

[5] Nash S, Desai S, Croxford S, et al Progress towards ending the HIV epidemic in the United Kingdom: 2018 report. London: Public Health England, 2018.

[6] Brown AE, Mohammed H, Ogaz D, et al Fall in new HIV diagnoses among men who have sex with men (MSM) at selected London sexual health clinics since early 2015: testing or treatment or pre-exposure prophylaxis (PreP)? Euro Surveill. 2017;22 10.2807/1560-7917.ES.2017.22.25.30553 PMC5490453 28662762

[7] World Health Organization Consolidated guidelines on the use of antiretroviral drugs for treating and preventing HIV infection 2016. Available: http://www.who.int/hiv/pub/arv/arv-2016/en/ 27466667

[8] Centers for Disease Control and Prevention Preexposure prophylaxis for the prevention of HIV infection in the United States, 2017 Available: https://www.cdc.gov/hiv/pdf/risk/prep/cdc-hiv-prep-guidelines-2017.pdf

[9] British HIV Organisation BHIVA/BASHH guidelines on the use of HIV pre-exposure prophylaxis (PreP), 2018 Available: https://www.bhiva.org/PrEP-guidelines

[10] European AIDS Clinical Society (EACS) EACS guidelines 9.0, 2017 Available: http://www.eacsociety.org/guidelines/eacs-guidelines/eacs-guidelines.html.

[11] Dolling DI, Desai M, McOwan A, et al An analysis of baseline data from the PROUD study: an open-label randomised trial of pre-exposure prophylaxis. Trials 2016;17 10.1186/s13063-016-1286-4 PMC4806447 27013513

[12] Rice BD, Yin Z, Brown AE, et al Monitoring of the HIV epidemic using routinely collected data: the case of the United Kingdom. AIDS Behav 2017;21(Suppl 1):83–90. 10.1007/s10461-016-1604-6 27832390

[13] Desai S, Nardone A, Hughes G, et al HIV incidence in an open national cohort of men who have sex with men attending sexually transmitted infection clinics in England. HIV Med 2017;18:615–22. 10.1111/hiv.12498 28127837

[14] Buchbinder SP, Glidden DV, Liu AY, et al HIV pre-exposure prophylaxis in men who have sex with men and transgender women: a secondary analysis of a phase 3 randomised controlled efficacy trial. Lancet Infect Dis 2014;14:468–75. 10.1016/S1473-3099(14)70025-8 24613084 PMC4133171

[15] Dunn DT, Gafos M, White E, et al HIV moments and pre-exposure prophylaxis – authors' reply. The Lancet 2016;387 10.1016/S0140-6736(16)00695-4 27115971

[16] Cambiano V, Miners A, Dunn D, et al Cost-effectiveness of pre-exposure prophylaxis for HIV prevention in men who have sex with men in the UK: a modelling study and health economic evaluation. Lancet Infect Dis 2018;18 10.1016/S1473-3099(17)30540-6 PMC5988036 29054789

[17] Ong KJ, Desai S, Field N, et al Economic evaluation of HIV pre-exposure prophylaxis among men-who-have-sex-with-men in England in 2016. Euro Surveill 2017;22 10.2807/1560-7917.ES.2017.22.42.17-00192 PMC5710117 29067902

[18] Poynten IM, Jin F, Prestage GP, et al Defining high HIV incidence subgroups of Australian homosexual men: implications for conducting HIV prevention trials in low HIV prevalence settings. HIV Med 2010;11:635–41. 10.1111/j.1468-1293.2010.00833.x 20456511

[19] Pathela P, Jamison K, Braunstein SL, et al Incidence and predictors of HIV infection among men who have sex with men attending public sexually transmitted disease clinics, New York City, 2007–2012. AIDS and Behavior 2017;21:1444–51. 10.1007/s10461-016-1499-2 27448826

[20] Grulich AE, Guy R, Amin J, et al Population-level effectiveness of rapid, targeted, high-coverage roll-out of HIV pre-exposure prophylaxis in men who have sex with men: the EPIC-NSW prospective cohort study. The Lancet HIV 2018;5:e629–37. 10.1016/S2352-3018(18)30215-7 30343026

