# Supplementary material for: Predictive factors for HIV infection among men who have sex with men and who are seeking PrEP: a secondary analysis of the PROUD trial
Source: Sex Transm Infect. 2019 Mar 27;95(6):449–54. doi: 10.1136/sextrans-2018-053808 (PMC6824743; doi:10.1136/sextrans-2018-053808)
Supplement: Supplementary data [file sextrans-2018-053808supp001.pdf]

Appendix

Table 1: Censoring method for HIV incidence analysis

Description of testing/follow-up	Censored:	N
HIV test after eligible for PrEP	Date of PrEP eligibility (first HIV test after week 48/October 2014)	205
HIV-positive during deferred phase	Date of first positive test result	21
Last HIV test before eligible for PrEP	Date of PrEP eligibility (week 48/October 2014)	19
Last HIV test at baseline	Date of PrEP eligibility (week 48/October 2014)	12
Initiated PrEP before eligible	Date of PrEP initiation	9
Co-enrollers	Date of randomisation to IMM arm	2
HIV-positive at baseline	Excluded from analysis	1
**Total**		**269**

Table 2: HIV incidence by number of sexual partners in 90 days prior to baseline visit

Characteristic		Number of participants (%)	Total PY	HIV infections	Incidence rate (per 100 PY)	95% CI	Rate ratio	95% CI	p-value*
**Number of AI partners**	1	24 (9.2)	22.3	1	4.5	0.1-24.9	0.3	0.01 -1.7	0.01
	2-4	47 (17.9)	41.7	0	0	0-8.9**	0.1	0-0.53**	
	5-9	49 (18.7)	43.8	4	9.1	2.5-23.4	0.6	0.2-1.8	
	10-19	70 (26.7)	61.7	6	9.7	3.6-21.2	0.6	0.2-1.7	
	20+	72 (27.5)	63.4	10	15.8	7.6-29.0	1.0	---	
**Number of IAI partners**	0	23 (8.9)	19.7	2	10.2	1.2-36.7	1.3	0.2-7.1	0.12
	1	45 (17.5)	41.4	2	4.8	0.6-17.4	0.6	0.1-3.4	
	2-4	55 (21.4)	48.6	2	4.1	0.5-14.9	0.5	0.1-2.9	
	5-9	54 (21.0)	49.6	4	8.1	2.2-20.6	1.0	---	
	10-19	45 (17.5)	37.9	5	13.2	4.3-30.8	1.6	0.4-6.8	
	20+	35 (13.6)	32.2	5	15.5	5.0-36.2	1.9	0.5-8.1	
**Number of RAI partners**	0	18 (7.0)	17.7	0	0	0-20.8**	0.6**	0-3.7**	0.02
	1	40 (15.5)	37.2	2	5.4	0.7-19.4	0.8	0.1-4.4	
	2-4	68 (26.4)	58.2	4	6.9	1.9-17.6	1.0	---	
	5-9	47 (18.2)	42.2	2	4.7	0.6-17.1	0.7	0.1-3.9	
	10-19	62 (24.0)	54.1	9	16.6	7.6-31.6	2.4	0.8-9.0	
	20+	23 (8.9)	20.1	3	14.9	3.1-43.6	2.2	0.4-10.5	
**Number of ncIAI partners**	0	37 (14.5)	32.3	3	9.3	1.9-27.2	2.9	0.4-24.3	0.03
	1	69 (27.1)	62.3	2	3.2	0.4-11.6	1.0	---	
	2-4	65 (25.5)	60.5	3	5.0	1.0-14.5	1.5	0.2-13.0	
	5-9	50 (19.6)	42.3	6	14.2	5.2-30.8	4.4	0.9-31.8	
	10+	34 (13.3)	30.0	6	20.0	7.3-43.6	6.2	1.3-44.9	
**Number of ncRAI partners**	0	32 (12.5)	29.8	1	3.4	0.1-18.7	1.2	0.04-16.0	0.01
	1	78 (30.5)	72.5	2	2.8	0.3-10.0	1.0	---	
	2-4	81 (31.6)	68.7	8	11.6	5.0-22.9	4.2	1.0-29.1	

	5-9	35 (13.7)	30.9	5	16.2	5.3-37.8	5.9	1.2-43.7	
	10+	30 (11.7)	25.7	4	15.6	4.2-39.9	5.7	1.0-44.1	
**Number of ncIAI HIV +ve partners**	0	142 (58.2)	128.0	9	7.0	3.2-13.4	1.0	---	0.04
	1	49 (20.1)	45.2	2	4.4	0.5-16.0	0.6	0.1-2.6	
	2-4	27 (11.1)	24.0	5	20.9	6.7-48.7	3.0	0.9-8.9	
	5-9	16 (6.6)	11.4	2	17.5	2.1-63.2	2.5	0.4-10.4	
	10+	10 (4.1)	9.1	2	22.0	2.7-79.4	3.1	0.5-13.1	
**Number of ncRAI HIV +ve partners**	0	141 (57.8)	128.1	9	7.0	3.2-13.3	1.0	---	0.03
	1	58 (23.8)	50.5	3	5.9	1.2-17.4	0.8	0.2-3.0	
	2-4	23 (9.4)	19.9	3	15.1	3.1-44.2	2.2	0.5-7.6	
	5-9	14 (5.7)	10.7	4	37.4	10.2-95.7	5.3	1.4-17.0	
	10+	8 (3.3)	7.4	1	13.4	0.3-74.8	1.9	0.1-11.6	

PY, person years; HIV, human immuno-deficiency virus; CI, confidence interval; RR, rate ratio; AI, anal intercourse; IAI, insertive anal intercourse; RAI, receptive anal intercourse; ncIAI, insertive anal intercourse without a condom; ncRAI, receptive anal intercourse without a condom;

Missing data (Total, events lost due to missing exposure data) for AI (6, 0); IAI (11, 1); RAI (10, 1); ncIAI (13, 1); ncRAI (12, 1); ncIAI with HIV +ve partner (24, 1); ncRAI with HIV +ve partner (24, 1).
*p-value for trend
**one-sided, 97.5% confidence interval
